# Executive Attention and Empathy-Related Responses in Boys with Oppositional Defiant Disorder or Conduct Disorder, With and Without Comorbid Anxiety Disorder

**DOI:** 10.1007/s10578-018-0810-z

**Published:** 2018-05-11

**Authors:** Jarla Pijper, Minet de Wied, Sophie van Rijn, Stephanie van Goozen, Hanna Swaab, Wim Meeus

**Affiliations:** 1 Department of Adolescent Development, Utrecht University, P.O. Box 80140, 3508 TC Utrecht, The Netherlands; 2 Clinical Child and Adolescent Studies, Leiden University, Leiden, The Netherlands; 3 School of Psychology, Cardiff University, Cardiff, UK; 4 Developmental Psychology, Tilburg University, Tilburg, The Netherlands

**Keywords:** Empathy, Sympathy, Oppositional defiant disorder, Conduct disorder, Emotion regulation, Executive attention

## Abstract

This is a first study that investigated the relationships between executive attention—as an important aspect of emotion regulation—and state empathy and sympathy in ODD/CD boys with (*N* = 31) and without (*N* = 18) comorbid anxiety disorder (7–12 years). Empathic reactions were evoked using three sadness-inducing film clips. One clip was highly evocative involving a bear cub losing his mother, whilst two other clips were mildly evocative involving children in common childhood situations. Self-reports of empathy and sympathy were collected and executive attention was assessed with a performance task. Poor executive attention skills were associated with less empathy and sympathy, particularly in ODD/CD boys with anxiety and under conditions of a highly evocative stimulus. Our findings support the view that different mechanisms may be involved in empathy problems of ODD/CD children.

## Introduction

Empathy problems have been associated with oppositional defiant disorder (ODD) and conduct disorder (CD) [1]. Children with ODD/CD constitute a heterogeneous group, however, and research suggests that there are individual differences in the mechanisms underlying empathy deficits in children with ODD/CD [1, 2]. Poor emotion regulation is common in children with ODD/CD [3, 4], and studies conducted with normal samples suggest that emotion regulation is a factor in empathy, especially in people who are highly sensitive to negative emotions [5]. Furthermore, studies with clinical samples show that ODD/CD boys exhibiting relatively high levels of anxiety (indicated by high resting heart rate) and poor emotional control (indicated by low levels of resting respiratory sinus arrhythmia) are selectively impaired in empathy-related responses with negative not positive emotions [6, 7]. Poor emotional control has been put forward as a factor that might be involved in their empathic responding [1]. Empirical evidence for this hypothesis is lacking, however. The current study takes up this issue and examines whether emotion regulation is involved in empathic responses to sadness inducing stimuli in boys with ODD/CD, with and without comorbid anxiety disorder.

Empathy, generally defined as the ability to understand and share another person’s feelings [8], is a complex construct, encompassing traits (a stable disposition), states (transient reactions), and affective and cognitive components [9]. Affective empathy is examined in this study, which refers to “the vicarious affective response to another person” [10, p. 29]. The literature often distinguishes empathy, sympathy and personal distress [11], although the constructs are closely related and often part of the same emotional experience. Empathy refers to a matching of emotions (feeling *with* the target), sympathy refers to feelings of sorrow or concern (feeling *for* the target), and personal distress is a self-focused aversive reaction evoked *by* the emotions of another person. Eisenberg et al. [12] hypothesized that individuals who cannot adequately regulate their emotions are more likely than others to become empathically over-aroused when they witness another person in distress, and to experience personal distress rather than sympathy. Well-regulated individuals are thought to maintain optimal levels of empathic arousal and to experience high levels of empathy/sympathy. This hypothesis has been confirmed by findings from numerous studies with children from community samples, using various measures of emotion regulation and empathy-related responses [5, 13, 14].

Eisenberg et al. [15] define emotion regulation as ‘processes used to manage and change if, when, and how (e.g., how intensely) one experiences emotions and emotion-related motivational and physiological states, as well as how emotions are expressed behaviorally’ (p. 288). One of these processes involved occurs at the cognitive level by deliberately shifting attention away from distressing stimuli and refocusing attention to positive or non-threatening ideas or objects. This deliberate use of attention processes is known as *executive attention* and can be subsumed under the larger construct of effortful control (a dimension of temperament related to the modulation of emotions and behaviour) [15–17]. Because executive attention could help regulating emotional reactivity, it is thought to be particularly important in the modulation of empathic arousal [18, 19].

Despite the suggested importance of executive attention in the regulation of empathic arousal, few empirical studies have addressed the relationship between executive attention and empathy or sympathy. In agreement with predictions, studies with community samples of school-aged children show positive relationships between behavioural measures of children’s executive attention and state indexes of sympathy concurrently [20] and over time with trait empathy/sympathy [21, 22]. The proposed relationships have further been supported by empirical studies demonstrating positive links between the broader construct of effortful control (e.g., inhibitory control) and indexes of trait empathy [23] and sympathy [24, 25] as well as with conscience development (related to sympathy) [26] in community samples.

The modulation of empathic arousal may be particularly important in highly sensitive people [5, 12], who are expected to be more susceptible for vicariously induced negative emotions. If they lack the ability to efficiently regulate themselves they may become overwhelmed, rendering them at risk for self-focused rather than other-focused attention and, in turn, feel lower levels of empathy/sympathy. Using different behavioural measures of emotion regulation, Eisenberg and her colleagues have put this hypothesis to the test in at least two studies with community samples of school-aged children [21, 22]. Inhibition, impulsivity and self-control were positively related to trait empathy/sympathy among children *high* in emotional sensitivity [21, 22], whilst executive attention was positively related to trait empathy/sympathy among those *low* in emotional sensitivity [22]. Results are thus still far from conclusive.

Children with ODD/CD constitute a heterogeneous group [27], including children who may show different neurocognitive dysfunctions associated with low threat sensitivity (e.g., those with callous-unemotional traits) or with high threat sensitivity [28]. Heightened threat sensitivity is thought to occur in ODD/CD children and adolescents without CU traits, and those with comorbid anxiety disorder. ODD/CD children with comorbid anxiety disorder may be highly susceptible for vicariously induced negative emotions, as they have been associated with hypersensitivity to perceived threat [28] and increased autonomic arousal [29]. For instance, male adolescent offenders with high levels of anxiety exhibited increased attentional bias to distressing stimuli than those with low levels of anxiety [30]. Furthermore, ODD/CD children with higher levels of anxiety exhibited higher cortisol stress reactivity than those with lower levels of anxiety [31], and ODD/CD boys with high rates of internalizing problems (including anxiety) had higher resting heart rate (HR) than normal controls [7, 32]. Moreover, children with ODD/CD have been found to show impairments in executive functions, including problems with attention shifting [33]. Consequently, the interplay between increased sensitivity to negative emotions and poor attentional regulation can render ODD/CD children with anxiety particularly susceptible to become over-aroused in response to other’s distress and show little empathy/sympathy for others, accordingly.

The main purpose of the present study was to examine whether executive attention is a factor in state empathy/sympathy in ODD/CD boys, with and without comorbid anxiety disorder. To evoke empathic reactions we used three sadness inducing film clips: one film clip portraying a bear cub in distress after his mother dies and two film clips involving children who experience sadness in common childhood situations [34]. In a prior study with ODD/CD boys, the bear clip was found to be more evocative than the human clips [34].

Theory [12] and empirical evidence [5] suggest that more efficient emotion regulation is associated with stronger empathy or sympathy. Therefore, we predicted that ODD/CD boys with stronger executive attention skills would report more empathy and sympathy. In addition, because aspects of emotion regulation are especially likely to be a factor in empathy among those highly sensitive to negative emotions [12] and under conditions of strong vicarious arousal [14], we expected the proposed relationship to be particularly evident within the group of ODD/CD boys with comorbid anxiety disorder and particularly under conditions of the most challenging stimulus (i.e., the bear clip). Finally, given the importance of CU traits in the ODD/CD literature [35], we investigated the effects of CU traits in the relationship between executive attention and empathic responses. Based on the assumption that heightened threat sensitivity is more likely to occur in ODD/CD children with low rather than high CU traits [28], we may expect a stronger role of emotion regulation in empathic responses amongst those with low rather than high CU traits.

## Method

This study was approved by the Medical Ethical Committee of Leiden University Medical Centre (LUMC), and parents gave written consent prior to participation according to the declaration of Helsinki.

### Recruitment and Participants

An initial group of 58 ODD/CD boys (7–12 years) was recruited via clinical health centers (*n* = 23) and special education (*n* = 35). The presence of psychopathology, as set out by the DSM-IV criteria, was determined by the parent-version of the Dutch Diagnostic Interview Schedule for Children (DISC-IV) [36]. Boys were excluded from the sample if they had an estimated intelligence quotient (IQ) below 70 (*n* = 3) or missed data on estimated IQ (*n* = 2). Estimated IQ was assessed with the subtests Block Design and Vocabulary of the Dutch Wechsler Intelligence Scale for Children (WISC-III-NL) [37]. Four additional participants were excluded from the sample because of missing data on executive attention (*n* = 2), CU traits (*n* = 1) and autism symptoms (*n* = 1). Autism symptoms were, among other variables, used to verify distinction between subgroups.

Our sample consisted of 49 ODD/CD boys (*M*_*age*_ = 10.35, *SD* = 1.27; *M*_*IQ*_ = 95.74, *SD* = 12.19). Those with comorbid anxiety disorder according to the DISC-IV (social phobia (*n* = 9), separation anxiety disorder (*n* = 10), specific phobia (*n* = 25), panic disorder (*n* = 1), agoraphobia (*n* = 1), generalized anxiety disorder (*n* = 9), obsessive compulsive disorder (*n* = 2), and/or post traumatic stress disorder (*n* = 3)) were assigned to the ODD/CD anxious group (ODD/CD + ANX), whereas those without were assigned to the ODD/CD non-anxious group (ODD/CD-ANX). The ODD/CD + ANX group consisted of 31 boys with ODD (*n* = 20) or CD (*n* = 11), of which 26 had comorbid attention deficit hyperactivity disorder (ADHD) and five comorbid depression. Eleven boys used psycho stimulants, one boy used anti-psychotics and one used both. The ODD/CD-ANX group consisted of 18 boys with ODD (*n* = 14) or CD (*n* = 4), of which nine had comorbid ADHD and one comorbid depression. Seven boys used psycho stimulants. Subgroups showed no differences in age or IQ. Further, no differences were obtained between boys with or without psycho-pharmacological treatment on main study variables.

### Stimuli

#### Empathy-Inducing Film Clips

To evoke empathic reactions, participants were exposed to three sadness-inducing film clips: one clip involving a girl (Anja) feeling depressed because she is ignored and badgered by her classmates (length: 57 s), one clip involving a boy (Mohammed) who fails at selection training for a soccer tournament (length: 148 s), and one clip involving a bear cub in distress after his mother dies (length 164 s). The clips have been used before to evoke empathic sadness in studies with ODD/CD boys [6] and adolescents [38]. A longer version of the bear clip (length: 256 s) was used in the study with ODD/CD boys mentioned above [34]. In the current study, a shorter version of the bear clip was used to keep in time with the human clips.

#### Relaxing Video

Because previous studies have documented that ODD/CD children with high rates of anxiety show increased autonomic arousal [7], we compared resting HR to verify distinction between subgroups. Prior to the empathy task, resting HR was assessed during a 5-min fragment from the video Coral Sea Dreaming (Small World Music, Inc.), found to promote relaxation [39] (for a detailed description of the assessment of HR, see [38]). Prior to each empathy clip, a 1-min fragment of the relaxing video was used to ensure recovery from emotional arousal induced by the previous clip.

#### Apparatus and Stimulus Presentation

Participants were individually tested in a laboratory room. All empathy-inducing clips were randomly presented on a 17-inch color monitor (Philips 109E50) that was placed on a desk. Following a 1-min aquatic video, the title of the clip (i.e., the name of the protagonist) appeared on the screen, and a female voice over introduced the clip. A program written with Delphi 6 controlled stimulus presentation and collected self-report data.

### Outcome Measures

#### Clinical Measures

To verify group distinction, subgroups were compared on level of (1) externalizing and internalizing problem behaviour, using the externalizing and internalizing scales (including subscales rule-breaking, aggressive behaviour, anxious/depressed, withdrawn/depressed and somatic complaints) of the Dutch *Child Behaviour Checklist (*CBCL*6-18*) [40], (2) CU traits, using combined parent and teacher ratings on the CU scale of the Dutch *Antisocial Process Screening Device* (APSD) [41], and (3) autism symptoms, using the total scale of the Dutch *Social Responsiveness Scale* (SRS) [42]. In the current study, internal consistency was acceptable for the externalizing (α = .89) and internalizing (α = .87) scales of the CBCL, poor for the CU scale (α = .56) of the APSD and good for the total scale of SRS (α = .94). Of all clinical measures, T-scores were used.

#### Empathy

Following each empathy clip, participants had to identify the *quality* and *intensity* of the emotions of the protagonists and their own experienced emotions. The quality was established by marking one or more cartoon faces visualizing fear, anger, happiness, sadness, surprise, or neutral (i.e., no emotion). The intensity was established by marking one out of four boxes increasing in size. If the participant identified the target emotion, the participant’s experienced (target) emotion was scored on a 5-point scale (0 = no empathy to 4 = very much). The participant received a 0 score if they incorrectly identified the target emotion(s), or did not experience the target emotion themselves. In response to the bear, all participants identified the target emotions (sadness or fear). In response to Mohammed 4 participants did not identify the target emotion (sadness) and in response to Anja 2 participants did not identify the target emotion (sadness). The correlation between the empathy scores obtained for Mohammed and Anja was significantly positive (*r* = .57). Both scores were combined to create one empathy score for the human clips, accordingly.

#### Sympathy

Following the empathy questions, participants were asked if they felt sorry for the protagonist by marking one of two boxes that represented yes or no. If they marked yes, participants were asked how much they felt sorry by marking one of four boxes increasing in size. Sympathy was scored on a 5-point scale (0 = no sympathy to 4 = very much). The sympathy scores of Mohammed and Anja were significantly related (*r* = .53), and were combined to create one sympathy score for the human clips.

#### Manipulation Check

The bear clip was thought to be more evocative than the human clips not only because of its theme, but also because cute young animals are likely to elicit strong positive feelings in children [43] and adults [44], which may minimize potential confounding effects of negative dispositions. In line with previous findings [6], a repeated measures ANOVA revealed that ODD/CD boys in the current study reported higher levels of empathy and sympathy to the bear clip than to the human clips, *F*(1,48) = 25.72, *p* < .001, *F*(1,48) = 68.03, *p* < .001, respectively. We therefore considered it legitimate to separately analyze the empathic responses of the bear clip and human clips.

#### Executive Attention

Executive attention was assessed with the *Shifting Attentional Set—Visual (SSV)* subtest of the Amsterdam Neuropsychological Tasks program [45]. The SSV measures attention shifting over three consecutive trials using a within task manipulation by correcting the recorded number of errors and reaction times of trial three from those of trial one. In the first trial, a constantly presented bar appears on the screen with a green block randomly jumping to the left or the right. The participant is instructed to follow the green block with the corresponding mouse key (left or right). In the second trial, a red block appears on the bar also randomly jumping to the left or the right. This time, however, the participant is instructed to respond in the opposite direction. In the third trial, the first and second trials are randomly mixed, which requires attention shifting. Although the SSV is primarily intended to measure attention shifting, it also taps into attention focusing and is therefore a good measure of executive attention. The SSV has been successfully employed in clinical samples [46, 47] and has satisfactory psychometric properties [48].

Number of errors and reaction time were significantly related (*r* = − .38). We controlled for reaction time in the analyses, revealing no significant influences to the findings. Accordingly, we only used number of errors in the analyses: more errors represented weaker executive attention.

### Procedures

Participants were invited to visit Leiden University for one day with one of their parents. During this day, parents signed informed consent, filled out questionnaires and completed the DISC-IV interview. Boys completed the empathy-task. Within 2 weeks, a second session took place either at the school or clinical health center of the participants. During this session, boys were administered to the subtests for estimated IQ and executive attention.

### Statistical Analyses

In *pre-analyses*, distinction between subgroups were verified by conducting independent samples t-tests, using ODD/CD subgroups as independent variable and scores on internalizing problem behaviour (anxious/depressed, withdrawal, somatic complaints), externalizing problem behaviour (aggressive, rule-breaking), CU traits and autism symptoms as well as resting levels of HR as dependent variables.

In *main analyses*, separate multiple hierarchical regression analyses were conducted with outcome measures empathy and sympathy of the bear clip, and empathy and sympathy of the human clips. In step 1, we entered group (coded as a dummy: 0 = ODD/CD-ANX, 1 = ODD/CD + ANX) and executive attention as predictors. In step 2, we entered the product term anxiety X executive attention to examine interaction effects. Significant interactions were dismantled by testing whether simple slopes were significantly different from zero for each subgroup [49]. To test for confounding effects of ADHD (DISC-IV diagnosis) and autism symptoms (SRS total score), regressions were re-analyzed with ADHD and autism symptom severity as covariates.

In *post-hoc* analyses, we re-analyzed the data with CU traits as a moderator. In addition, because of a potential lack of power due to our small sample size, we checked the main significant findings with bootstrap analyses by drawing 1000 resamples from our original sample [50].

Prior to regression analyses variables were standardized (*M* = 0; *SD* = 1). Probabilities of all tests were two-tailed using a significance level of 0.05.

## Results

### Pre-analyses

There were no differences between the subgroups in externalizing problems, with both groups scoring in the clinical range of the externalizing problem behaviour scale (T > 60). As expected, the ODD/CD + ANX group had significantly higher scores on the CBCL internalizing scale than the ODD/CD-ANX group; the ODD/CD + ANX group scored in the clinical range (T > 60), whereas the ODD/CD-ANX group scored in the normal range. Further, the ODD/CD + ANX group had significantly higher resting HR and significantly more autism symptoms than ODD/CD-ANX, even though both scored in the moderate range (60 < T < 74) [42]. Unexpectedly, however, no differences were obtained between subgroups in terms of CU traits; both scored in the slightly atypical range (56 < T < 60) [51]. Overall, these results confirm the distinction between ODD/CD boys with and without anxiety. Table 1 represents an overview of *M* and *SD* of the clinical measures and resting HR.

**Table 1:** Verification of subgroups

	ODD/CD -ANX (*n* = 18)		ODD/CD + ANX (*n* = 31)		t-values (df = 47)
	*M*	*SD*	*M*	*SD*	
Resting HR	77.11	10.23	83.88	10.03	− 2.26*
CBCL					
Internalizing^a^	55.50	9.89	69.23	6.70	− 5.23***
Anxious/depressed	56.39	7.66	68.74	7.91	− 5.33***
Withdrawn/depressed	60.89	7.13	67.65	8.34	− 2.88**
Somatic complaints	54.17	5.58	62.71	8.76	− 3.71**
Externalizing	66.61	9.88	67.81	8.32	− .45
Rule breaking	62.94	7.22	61.71	7.45	.57
Aggressive	70.17	10.92	71.16	10.59	− .31
CU traits	58.78	6.91	58.26	9.46	.20
Autism symptoms	61.78	11.56	73.45	13.81	− 3.02*

*df* degrees of freedom, *IQ* intelligence quotient, *CBCL* child behavior checklist, *HR* heart rate, *CU* callous unemotional

*p < .05, **p < .01, ***p < .001

^a^df = 26.21

### Main Analyses

Table 2 represents *M* and *SD* of main study variables and Table 3 represents bivariate correlations between main study variables.

**Table 2:** Main study variables: M and SD (total sample and subgroups)

	Total sample		ODD/CD-ANX		ODD/CD + ANX		t-values (df = 47)
	*M*	*SD*	*M*	*SD*	*M*	*SD*	
1. Empathy human^a^	.88	1.16	.39	.72	1.18	1.28	− 2.58**
2. Sympathy human	2.26	1.22	2.11	1.32	2.35	1.16	− .67
3. Empathy bear	2.00	1.67	1.56	1.69	2.26	1.63	− 1.43
4. Sympathy bear	3.37	.88	3.11	.96	3.52	.81	− 1.57
5. Executive attention	3.39	4.84	2.06	2.58	4.16	5.66	− 1.78
6. CU traits	6.86	2.25	6.94	1.83	6.81	2.50	.20

*CU* callous unemotional raw scores

**p < .01

^a^df = 46.98

**Table 3:** Bivariate correlations between main study variables (total sample)

	1	2	3	4	5
1. Empathy human					
2. Sympathy human	.43**				
3. Empathy bear	.46**	.38**			
4. Sympathy bear	.40**	.65**	.55**		
5. Executive attention	− .12	− .09	− .26	− .22	
6. CU traits	− .06	− .29*	− .11	− .13	.29*

Correlations were based on standardized scores

*CU* callous unemotional

*p < .05, **p < .01

#### Bear Clip

In relation to *empathy*, Table 4 shows that executive attention was a significant negative predictor, *F*(2,46) = 3.74, *p* = .031. This suggests that weaker executive attention skills (i.e., more errors on the task) were associated with less empathy. Group was not a significant positive predictor of empathy. Importantly, a significant interaction was obtained between executive attention and anxiety, *F*(3,45) = 5.05, *p* = .004. In the ODD/CD + ANX subgroup, executive attention was negatively associated with empathy (B = − .44, SE = .14, *p* = .003), whereas no such association was found in the ODD/CD-ANX subgroup (B = .68, SE = .41, *p* = .102). Model’s adjusted R square was .20, representing a small to moderate effect.

**Table 4:** Group and executive attention predicting empathic responses of the bear clip and human clips

Predictor	Bear clip				Human clips			
	Empathy		Sympathy		Empathy		Sympathy	
	β	*R*^2^∆	β	*R*^2^∆	β	*R*^2^∆	β	*R*^2^∆
Step 1		.14*		.12*		.15*		.02
Executive attention	− .32*		− .28		− .20		− .11	
Group	.27		.28		.37*		.12	
Step 2		.11*		.07*		.00		.04
Group X executive attention	− 1.04*		− .84*		− .18		− .64	
Total *R*^2^		.25		.20		.15		.07

The findings remained the same after controlling for ADHD and autism symptoms

^*^p < .05

In relation to *sympathy*, Table 4 shows that executive attention and group were not significant predictors, although the overall model was significant, *F*(2,46) = 3.27, *p* = .047. Importantly, a significant interaction was found between executive attention and group, *F*(3,45) = 3.69, *p* = .018. Again, further analyses revealed that in the ODD/CD + ANX subgroup executive attention was negatively associated with sympathy (B = − .37, SE = .14, *p* = .013), whereas no such association was found in the ODD/CD-ANX subgroup (B = .53, SE = .42 *p* = .216). Model’s adjusted R square was .14, representing a small to moderate effect.

#### Human Clips

In relation to *empathy*, Table 4 shows that executive attention was not a significant predictor, whereas group was a significant positive predictor, *F*(2,46) = 3.99, *p* = .025. The ODD/CD + ANX subgroup reported more empathy than the ODD/CD-ANX subgroup. There was no significant interaction between group and executive attention. In relation to *sympathy*, no significant predictors or interaction were found.

### Post-hoc Analyses

#### CU Traits as a Moderator in Human Clips and Bear Clip

Interestingly, a significant interaction was found between executive attention and CU traits (β = .37, *p* = .018) in relation to empathy (not sympathy; β = .04, n.s.) for the human clips, though the overall model was not significant, *R*^2^ = .13, *F*(3,45) = 2.30, *p* = .091. At low levels of CU traits, executive attention was significantly negatively associated with empathy (B = − .57, SE = .24, *p* = .019), whereas no such association was found at moderate (B = − .22, SE = .15, n.s.) to high (B = .12, SE = .17, n.s.) levels of CU traits. Model’s adjusted R square was .08, representing a small effect. No other significant effects emerged with respect to the human clips (beta’s ranged from − .29 to .00, *p*-values > .06), nor to the bear clip (beta’s ranged from − .25 to .16., *p*-values > .10).

#### Bootstrap Analyses

Bootstrap analyses supported main significant findings; that is, significant interactions between executive attention and group in relation to empathy (B = − 1.12, SE = .43, bootstrapped SE = .45 and 95% CI − 1.67 to .22) and sympathy (B = − .90, SE = .45, bootstrapped SE = .54 and 95% CI − 1.77 to .56) of the bear clip as well as the interaction between executive attention and CU traits in relation to empathy of the human clips (B = .35, SE = .14, bootstrapped SE = .15 and 95% CI .11–.72), demonstrating limited statistical biases between estimates.

## Discussion

The current study examined whether executive attention is a factor in empathic responses to sadness inducing film clips amongst ODD/CD boys, with and without comorbid anxiety disorder. In agreement with predictions, executive attention was found to relate significantly to empathy and sympathy, such that those with poor executive attention skills reported less empathy and sympathy. The proposed relationships were evident only under conditions of a highly evocative stimulus (the bear clip) and only among ODD/CD boys with comorbid anxiety disorder. As such, our findings are in agreement with prior evidence that aspects of emotion regulation are associated with empathic responses particularly in highly sensitive children [12, 21, 22]. Our findings imply that strong executive attention is beneficial for ODD/CD children with anxiety disorder, as it may help to maintain optimal levels of empathic arousal and higher levels of empathy/sympathy, accordingly. Poor executive attention, on the other hand, may render them particularly susceptible for empathic over-arousal and personal distress—a self-focused emotion—rather than empathy/sympathy. Indeed, prior studies have demonstrated self-focused behaviour in reaction to other’s negative emotions in fearful children [52, 53], and inadequate regulation strategies in children with anxiety disorder [54].

Children with ODD/CD who also meet criteria of anxiety disorder may show heightened threat sensitivity because of an overly responsive *basic threat circuit* (amygdala, hypothalamus, periaqueductal grey) [28, 35]. This over-responsiveness is thought to result from early trauma or inadequate regulation. There is indeed empirical evidence indicating that ODD/CD individuals with comorbid mood and anxiety conditions show increased reactivity in neural regions involving the threat circuit (e.g., amygdala) and reduced reactivity in neural regions involving the regulation of the circuit (e.g., ventromedial frontal cortex) [55]. Abnormalities in these regions may be associated with general difficulties in the processing of emotional expressions [56, 57], which may influence empathy development, accordingly.

Under conditions of mild evocative stimuli (human clips), ODD/CD boys with anxiety reported more empathy than those without anxiety. Children with anxiety have been linked to bully victimization and social exclusion [58]. Given the content of the emotional stimuli (i.e., a girl being bullied and a boy being excluded from a soccer team), ODD/CD boys with anxiety may have been able to better identify themselves with the targets, leading to more empathy compared to ODD/CD boys without anxiety, accordingly. Alternatively, the content of the stimuli could have induced aversive arousal and more personal distress in those with than without anxiety. Empathy and personal distress are highly related constructs often stemming from the same emotional event [11]. The self-reports of empathy could have partly captured feelings of personal distress.

Because it is has also been proposed that heightened threat sensitivity is more likely to be present in ODD/CD children with low rather than high CU traits [28, 35], we expected a stronger effect of emotion regulation in empathic responses amongst those with low rather than high CU traits. In agreement with these expectations, we found preliminary evidence that weaker executive attention skills were significantly related to less empathy in ODD/CD boys with low levels of CU traits but not in those with moderate to high levels of CU traits.

Because ODD/CD children with high levels of CU traits are more likely to belong to the “fearless” rather than “fearful” subtype [59], one would expect higher levels of CU traits in ODD/CD boys without anxiety than in those with anxiety. Nevertheless, in the current study there were no significant differences in CU traits between those with and without comorbid anxiety disorder. This finding is in line with growing evidence that anxiety and CU traits are not necessarily related or mutually exclusive [30, 60, 61]. This evidence, together with the current finding of distinct moderating effects of anxiety and CU traits suggests that studies of empathy in ODD/CD children need to include both anxiety and CU traits measures.

Strengths of our study can be seen in the inclusion of a well-defined sample of ODD/CD children; those with anxiety had higher resting HR and higher scores on indexes of internalizing problems than those without anxiety. Further strengths are the standardized measurements of empathic responses and executive attention. However, some important limitations should be taken into consideration while interpreting our findings. First, we only focused on executive attention, whereas prior community-sample studies have also linked other indexes of emotion regulation to empathic responses, such as impulsivity [24] and physiological regulation [62]. Second, we did not include a measure of personal distress, thereby only partly testing Eisenberg and colleagues’ hypothesis regarding the role of emotion regulation in empathic responses [12]. Third, there is some evidence that animal-directed empathy does not necessarily transfer to human-directed empathy [63]. We should therefore be careful in generalizing the role of executive attention in the modulation of strong vicarious arousal to an inter-human empathy context. Fourth, in the current study we only investigated boys. This limits the generalizability of our findings to girls, as prior studies have shown empathy differences between boys and girls [64]. Fifth, the CU scale of the APSD showed poor internal consistency. However, poor internal consistency has previously been found for the CU scale [65], and in a prior study conducted with the current ODD/CD sample it showed good concurrent validity with the externalizing scale of the Teacher Report From [66]. Finally, we investigated a small sample with limit statistical power.

Our findings offer promising new research leads. For instance, it would be interesting to replicate our findings with physiological markers of emotion regulation and empathic responses. Physiological regulation has been linked to empathic responses in normal children [62], but not yet in children with ODD/CD. In addition, physiological markers of empathic responses could provide an index for personal distress (i.e., heart rate acceleration to emotional stimuli) [67].

If replicated, the findings of this study could provide important implications for clinical practice. Training attention in children with autism improved their academic performance [68] and clinical outcomes in children with anxiety disorders [e.g., 69, 70]. We therefore speculate that training executive attention in ODD/CD children with poor executive attention might be an initial step towards improving their empathic functioning.

This study provides initial evidence that executive attention is associated with empathic responses under conditions of highly evocative stimuli, especially in ODD/CD boys with comorbid anxiety disorder. It supports current ideas that different mechanisms may be involved in empathy problems in children and adolescents with ODD or CD.

## Summary

Children with ODD/CD are thought to have little empathy [1, 35]. This is an initial study that investigated the hypothesis [1] that emotion regulation is a factor in empathy deficits of children with ODD/CD. In agreement with expectations, our findings revealed that poor executive attention—as an important aspect of emotion regulation—was associated with lower levels of empathic responses, particularly in ODD/CD boys with comorbid anxiety disorder and only under conditions of a highly evocative stimulus. These findings imply that, in the presence of strong vicarious arousal, ODD/CD boys with anxiety and poor executive attention may be unable to maintain optimal levels of empathic arousal. Rather, they may experience empathic over-arousal, become self-focused instead of other-focused, and feel less empathy/sympathy for another person in distress, accordingly. Strengthening their executive attention skills may be an initial step towards improving their empathic responding. The current study highlights that children with ODD/CD form a heterogeneous group and that there may be individual differences in the mechanisms underlying their empathy deficits.

## References

[1] De Wied M, Gispen-De Wied C, Van Boxtel A (2010). Empathy dysfunction in children and adolescents with disruptive behavior disorders. Eur J Pharmacol.

[2] Pijper J, De Wied M, Van Goozen S, Meeus W, Centifanti LC, Williams DM (2017). Empathy problems in youth with disruptive behavior disorders, with and without callous unemotional traits. The Wiley handbook of developmental psychopathology.

[3] Matthys W, Vanderschuren LJMJ, Schutter DJLG, Lochman JE (2012). Impaired neurocognitive functions affect social learning processes in oppositional defiant disorder and conduct disorder: implications for interventions. Clin Child Fam Psychol Rev.

[4] Hobson CW, Scott S, Rubia K (2011). Investigation of cool and hot executive function in ODD/CD independently of ADHD. J Child Psychol Psychiatry.

[5] Eisenberg N, Fabes RA, Spinrad TL, Damon W, Lerner RM, Eisenberg N (2006). Prosocial behavior. Handbook of child psychology: Vol.3. Social, emotional, and personality development.

[6] De Wied M, Van Boxtel A, Zaalberg R, Goudena PP, Matthys W (2006). Facial EMG responses to dynamic emotional facial expressions in boys with disruptive behavior disorders. J Psychiatry Res.

[7] De Wied M, Van Boxtel A, Posthumus JA, Goudena PP, Matthys W (2009). Facial EMG and heart rate responses to emotion-inducing film clips in boys with disruptive behavior disorders. Psychophysiology.

[8] Eisenberg N, Strayer J, Eisenberg N, Strayer J (1987). Critical issues in the study of empathy. Empathy and its development.

[9] Davis MH (1996). Empathy: a social psychological approach.

[10] Hoffman ML (2000). Empathy and moral development: implications for caring and justice.

[11] Eisenberg N, Eggum ND, Decety J, Ickes W (2009). Empathic responding: sympathy and personal distress. The social neuroscience of empathy.

[12] Eisenberg N, Fabes RA, Murphy B, Karbon M, Maszk P, Smith M, O’Boyle C, Suh K (1994). The relations of emotionality and regulation to dispositional and situational empathy-related responding. J Pers Soc Psychol.

[13] Eisenberg N (2000). Emotion, regulation, and moral development. Annu Rev Psychol.

[14] Eisenberg N, Mikulincer M, Shaver PR (2010). Empathy-related responding: links with self-regulation, moral judgement and moral behavior. Prosocial motives, emotions and behavior: the better angels of our nature.

[15] Eisenberg N, Hofer C, Vaughan J, Gross JJ (2007). Effortful control and its socioemotional consequences. Handbook of emotion regulation.

[16] Eisenberg N, Smith CL, Spinrad TL, Vohs KD, Baumeister RF (2011). Effortful control: relations with emotion regulation, adjustment, and socialization in childhood. Handbook of self-regulation: research, theory and applications.

[17] Rothbart MK, Rueda MR, Mayr U, Awh E, Keele S (2005). The development of effortful control. Developing individuality in the human brain: a tribute to Michael I. Posner.

[18] Eisenberg N, Fabes RA, Nyman M, Bernzweig J, Pinuelas A (1994). The relations of emotionality and regulation to children’s anger-related reactions. Child Dev.

[19] Eisenberg N, Sulik MJ (2012). Emotion-related self-regulation in children. Teach Psychol.

[20] Gurthrie IK, Eisenberg N, Fabes RA, Murphy BC, Holmgren R, Mazsk P, Suh K (1997). The relations of regulation and emotionality to children’s situational empathy-related responding. Motiv Emot.

[21] Eisenberg N, Fabes RA, Murphy B, Karbon M, Smith M, Maszk P (1996). The relations of children’s dispositional empathy-related responding to their emotionality, regulation, and social functioning. Dev Psychol.

[22] Eisenberg N, Fabes RA, Shepard SA, Murphy BC, Jones S, Guthrie IK (1998). Contemporaneous and longitudinal prediction of children’s sympathy from dispositional regulation and emotionality. Dev Psychol.

[23] Rothbart MK, Ahadi SA, Hershey KL (1994). Temperament and social behavior in childhood. Merrill Palme.

[24] Eisenberg N, Michalik N, Spinrad TL, Hofer C, Kupfer A, Valiente C (2007). The relations of effortful control and impulsivity to children’s sympathy: a longitudinal study. Cogn Dev.

[25] Murphy BC, Shepard SA, Eisenberg N, Fabes RA, Guthrie IK (1999). Contemporeneous and longitudinal relations of dispositional sympathy to emotionality, regulation, and social functioning. J Early Adolesc.

[26] Kochanska G, Knaack A (2003). Effortful control as a personality characteritic of young children: antecedents, correlates, and consequences. J Pers.

[27] Frick PJ, Ray JV, Thornton LC, Kahn RE (2013). Can callous-unemotional traits enhance the understanding, diagnosis, and treatment of serious conduct problems in children and adolescents? A comprehensive review. Psychol Bull.

[28] Blair RJR, Leibenluft E, Pine DS (2014). Conduct disorder and callous-unemotional traits in youth. N Engl J Med.

[29] Bubier JL, Drabick DAG (2009). Co-occurring anxiety and disruptive behavior disorders: the roles of anxious symptoms, reactive aggression, and shared risk processes. Clin Psychol Rev.

[30] Kimonis ER, Frick PJ, Cauffman E, Goldweber A, Skeem J (2012). Primary and secondary variants of juvenile psychopathy differ in emotion processing. Dev Psychopathol.

[31] Van Goozen SHM, Matthys W, Cohen-Kettenis PT, Buitelaar JK, Van Engeland H (2000). Hypothalamic-pituitary-adrenal axis and autonomic nervous system activity in disruptive children and matched controls. J Am Acad Child Adolesc Psychiatry.

[32] Lahey BB, Loeber R, Burke J, Rathouz PJ, McBurnett K (2002). Waxing and waning in concert: dynamic comorbidity of conduct disorder with other disruptive and emotional problems over 17 years among clinic-referred boys. J Abnorm Child Psychol.

[33] Ter-Stepanian M, Grizenko N, Cornish K, Talwar V, Mbekou V, Schmitz N, Joober R (2016). Attention and executive function in children diagnosed with attention deficit hyperactivity disorder and comorbid disorders. J Can Acad Child Adolesc Psychiatry.

[34] De Wied M, Goudena PP, Matthys W (2005). Empathy in boys with disruptive behavior disorders. J Child Psychol Psychiatry.

[35] Blair RJR (2013). The neurobiology of psychopathic traits in youths. Nat Rev Neurosci.

[36] Ferdinand RF, Van der Ende J (2002). NIMH-DISC-IV: diagnostic interview schedule for children (Authorized Dutch version).

[37] Kort W, Schittekatte M, Dekker PH, Verhaege P, Compaan EL, Bosmans M (2005). Manual wechsler intelligence scale for children—Dutch edition (WISC-III-NL).

[38] De Wied M, Van Boxtel A, Matthys W, Meeus W (2012). Verbal, facial and autonomic responses to empathy-eliciting film clips by disruptive male adolescents with high versus low callous-unemotional traits. J Abnorm Child Psychol.

[39] Piferi RL, Kline KA, Younger J, Lawler KA (2000). An alternative approach for achieving cardiovascular baseline: viewing an aquatic video. Int J Psychophysiol.

[40] Verhulst FC, Van der Ende J (2013). Handleiding ASEBA-Vragenlijsten voor leeftijden 6 t/m 18 jaar. CBCL/6–18, YSR en TRF.

[41] De Wied M, Van der Baan H, Raaijmakers Q, De Ruiter C, Meeus W (2014). Factor structure and construct validity of the Dutch version of the antisocial process screening device. J Psychopathol Behav Assess.

[42] Roeyers H, Thys M, Druart C, De Schryver M, Schittekatte M (2011). Screeningslijst voor autismespectrumstoornissen: handleiding.

[43] Endenburg N (1995). The attachment of people to companion. Anthrozoös.

[44] Archer J, Monton S (2011). Preferences for infant facial features in pet dogs and cats. Ethology.

[45] De Sonneville LML (2014). Handboek Amsterdamse neuropsychologische taken.

[46] Jahja R, Huijbregts SCJ, de Sonneville LMJ, van der Meere JJ, van Spronsen FJ (2014). Neurocognitive evidence for revision of treatment targets and guidelines for phenylketonuria. J Pediatr.

[47] Van Rijn S, Swaab H (2015). Executive dysfunction and the relation with behavioral problems in children with 47,XXY and 47,XXX. Genes. Brain Behav.

[48] De Sonneville LMJ (2005). Amsterdam Neuropsychological Tasks: scientific and clinical applications. Tijdschrift Voor Neuropsychologie.

[49] Hayes AF, Matthes J (2009). Computational procedures for probing interactions in OLS and logistic regression: SPSS and SAS implementations. Behav Res Methods.

[50] Davison AC, Hinkley DV (1997). Bootstrap methods and their application.

[51] Frick PJ, Hare RD (2001). Antisocial process screening device (APSD).

[52] Liew J, Eisenberg N, Spinrad TL, Eggum ND, Haugen RG, Kupfer A (2011). Physiological regulation and fearfulness as predictors of young children’s empathy-related reactions. Soc Dev.

[53] Spinrad TL, Stifter CA (2006). Toddlers’ empathy-related responding to distress: predictions from negative emotionality and maternal behavior in infancy. Infancy.

[54] Legerstee JS, Garnefski N, Jellesma FC, Verhulst FC, Utens EMWJ (2010). Cognitive coping and childhood anxiety disorders. Eur Child Adolesc Psychiatry.

[55] Crowe SL, Blair RJR (2008). The development of antisocial behavior: what can we learn from functional neuroimaging studies?. Dev Psychopathol.

[56] Coccaro EF, McCloskey MS, Fitzgerald DA, Phan KL (2007). Amygdala and orbitofrontal reactivity to social threat in individuals with impulsive aggresion. Biol Psychiatry.

[57] Park G, van Bavel JJ, Vasey MW, Thayer JF (2013). Cardiac vagal tone predicts attentional engagement to and disengagement from fearful faces. Emotion.

[58] Bifulco A, Schimmenti A, Jacobs C, Bunn A, Rusu AC (2014). Risk factors and psychological outcomes of bullying victimization: a community-based study. Child Ind Res.

[59] Raine A (1993). The psychopathology of crime: criminal behavior as a clinical disorder.

[60] Fanti KA, Demetriou CA, Kimonis ER (2013). Variants of callous-unemotional conduct problems in a community sample of adolescents. J Youth Adolesc.

[61] Kahn RE, Frick PJ, Golmaryami FN, Marsee MA (2017). The moderating role of anxiety in the associations of callous-unemotional traits with self-report and laboratory measures of affective and cognitive empathy. J Abnorm Child Psychol.

[62] Taylor ZE, Eisenberg N, Spinrad TL (2015). Respiratory sinus arrhythmia, effortful control, and parenting as predictors of children’s sympathy across early childhood. Dev Psychol.

[63] McPhedran S (2009). A review of the evidence for associations between empathy, violence, and animal cruelty. Aggress Violent Behav.

[64] Van der Graaff J, Branje S, De Wied M, Hawk S, Van Lier P, Meeus W (2014). Perspective taking and empathic concern in adolescence: gender differences in developmental changes. Dev Psychol.

[65] Pardini DA, Lochman JE, Frick PJ (2003). Callous/unemotional traits and social-cognitive processes in adjudicated youths. J Am Acad Child Psy.

[66] Pijper J, De Wied M, VanVan RijnGoozen S, Swaab H, Meeus M (2016). Callous unemotional traits, autism spectrum disorder symptoms and empathy in boys with oppositional defiant disorder or conduct disorder. Psychiatry Res.

[67] Eisenberg N, Fabes RA (1990). Empathy: conceptualization, measurement, and relation to prosocial behavior. Motiv Emot.

[68] Spaniol MM, Shalev L, Kossyvaki L, Mevorach C (2018). Attention training in autism as a potential approach to improving academic performance: a school-based pilot study. J Autism Dev Disord.

[69] Bar-Haim Y, Morag I, Glickman S (2011). Training anxious children to disengage attention from threat: a randomized controlled trial. J Child Psychol Psychiatry.

[70] Bechor M, Pettit W, Silverman WK, Bar-Heim Y, Abend R, Pine DS, Vasey MW, Jaccard J (2014). Attention bias modification treatment for children with anxiety disorders who do not respond to cognitive behavioral therapy: a case series. J Anxiety Disord.

